# Isolation of an imported subgenotype B5 strain of human enterovirus A71 in Chongqing City, China, 2014

**DOI:** 10.1186/s12985-016-0571-x

**Published:** 2016-06-29

**Authors:** Qian Yang, Yong Zhang, Dongmei Yan, Shuangli Zhu, Dongyan Wang, Tianjiao Ji, Wei Huang, Hongqiu An, Wenbo Xu

**Affiliations:** WHO WPRO Regional Polio Reference Laboratory and Key Laboratory of Medical Virology and Viral Disease, Ministry of Health, National Institute for Viral Disease Control and Prevention, Chinese Center for Disease Control and Prevention, Beijing, 102206 People’s Republic of China; Chongqing Center for Disease Control and Prevention, Chongqing, 400042 People’s Republic of China

**Keywords:** Human enterovirus A71, Subgenotype B5, Importation

## Abstract

**Background:**

Enterovirus A71 (EV-A71) is the main pathogen responsible for large outbreaks of hand, foot, and mouth disease (HFMD) in mainland China, and the dominant EV-A71 strains belong to subgenotype C4. To date, only one imported subgenotype B5 of EV-A71 has been reported in Xiamen City Fujian Province, 2009.

**Results:**

Here, we report on another imported subgenotype B5 of EV-A71 isolated from a HFMD patient in Chongqing City in 2014 (strain CQ2014-86/CQ/CHN/2014, hereafter refer as CQ2014-86). The *VP1* coding sequence and the whole genome sequence revealed that strain CQ2014-86 shares the high nucleotide identity with Vietnamese strains isolated in 2011–2013, suggesting that strain CQ2014-86 may have been imported from Vietnam. In the 5’UTR, P2 and P3 regions, recombination events were found between strain CQ2014-86 and other EV-A, such as coxsackievirus A4 (CV-A4), CV-A5, CV-A14 and CV-A16.

**Conclusions:**

This is the second report on importation of subgenotype B5 of EV-A71 in China, implying that we need to pay more attention to the importation of different subgenotypes of EV-A71.

## Background

Human enterovirus A71 (EV-A71) is a neurotropic pathogen that can cause severe hand, foot, and mouth disease (HFMD) in children under 5 years old. It belongs to species EV-A (genus *enterovirus,* family *picornaviridae,* order *Picornavirales)*. Following large scale outbreaks of HFMD in 2008 in mainland China, it has been categorized “C” group notifiable infectious diseases by the Ministry of Health of China [1–3]. Since then, China has built a HFMD laboratories network across the whole country, and this network is crucial for monitoring the prevalence of EV serotypes associated with HFMD patients, especially EV-A71. The molecular epidemiology of EV-A71 in mainland China reflects the pattern of circulation of subgenotype C4 viruses. In this study, we report on an imported subgenotype B5 of EV-A71 isolated from a HFMD patient in Chongqing City, China, in 2014.

## Methods

In total, 210 clinical samples were collected in Chongqing City in 2014. Viruses were isolated from the original clinical specimens by propagation in human rhabdomyosarcoma (RD) and human larynxarcinoma (HEp-2) cells by conventional methods. Viral RNA was extracted using QIAamp Viral RNA mini kit (QIAGEN, Germany). Real-time reverse transcription-polymerase chain reaction [4], molecular typing and phylogenetic analysis was performed as described previously [2]. And strain CQ2014-86 was identified as subgenotype B5 of EV-A71.

The viral RNA was converted to cDNA by a random priming strategy. The cDNA was amplified using primers according to the previous report [5], and a primer-walking strategy was used to close the gaps as necessary. PCR products were purified for sequencing using a QIAquick Gel Extraction Kit (QIAGEN), after which the amplicons were sequenced bidirectionally using fluorescent dideoxy-chain terminators and an ABI PRISM 3130 Genetic Analyzer (Applied Biosystems, Foster City, CA, USA). The 5′-segment sequence was determined using a 5′-Rapid Amplification of cDNA Ends Core Set (Takara Biomedicals, Dalian, China), according to the manufacturer’s instructions.

The nucleotide sequences of EV-A71 strains was aligned using Bioedit sequence alignment editor software (version 5.0). Maximum likelihood (ML) trees were constructed using the best-fit Kimura 2-parameter + I model of nucleotide substitution in Mega software (version5.03) [6]. Similarity plot and bootscanning analyses were performed using the Simplot program (version 3.5.1; Stuart Ray, Johns Hopkins University, Baltimore, MD, USA).

## Results

Not surprisingly, all EV-A71 strains belonged to subgenotype C4a except one strain, strain CQ2014-86/CQ/CHN/2014 (hereafter refer as strain CQ2014-86), which was identified as subgenotype B5. Until now, only one whole genome sequence of subgenotype B5 from mainland China has been published (JN964686-Xiamen/FJ/CHN/2009) [5]. Here, we report the full-length genome sequence of a subgenotype B5 of EV-A71 (strain CQ2014-86) isolated in Chongqing City, China, during HFMD surveillance. The sequence of strain CQ2014-86 has been deposited in the GenBank database under the accession number KU647000. Strain CQ2014-86 was isolated from a throat swab specimen from a 4-year-old girl. The genome length of CQ2014-86 is 7412 nucleotides. Similarity between the only two strains that have full-length genomic sequences (Chongqing and Xiamen strains) isolated in mainland China is 95.75 % at the nucleotide level and 98.49 % at the amino acid level.

**Fig. 1:**
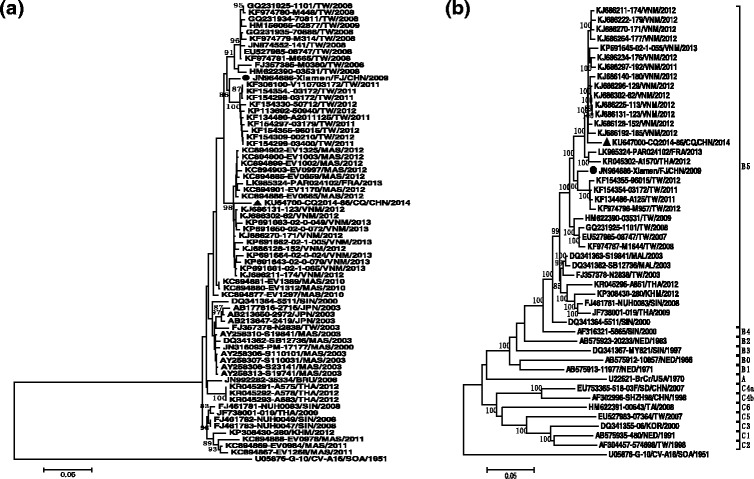
**a** Phylogenetic tree based on the entire VP1 coding region (891 bp) of subgenotype B5 of EV-A71. Black triangle indicated Chongqing strain KU647000-CQ2014-86/CQ/CHN/2014. Black round indicated Xiamen strain JN964686-Xiamen/FJ/CHN/2009. Strain CQ2014-86 and reference strains from GenBank database of subgenotype B5 of EV-A71 were included. Neighbor-joining algorithm was used for phylogenetic analysis with Mega software (version5.03). **b** Phylogenetic tree based on the complete genome of subgenotype B5 of EV-A71. Black triangle indicated Chongqing strain KU647000-CQ2014-86/CQ/CHN/2014. Black round indicated Xiamen strain JN964686-Xiamen/FJ/CHN/2009. The prototype of EV-A71(BrCr), CV-A16(G-10) and the oldest sequences of different subgenotypes of EV-A71 (B0-5, C1-6 subgenotypes) were included as well

Phylogenetic analysis was conducted based on the entire *VP1* coding region of Chongqing subgenotype B5 of EV-A71 sequence and other subgenotype B5 of EV-A71 sequences from the GenBank database (68 strains) (Fig. 1a). In addition, a phylogenetic tree based on the complete genome sequence of subgenotype B5 of EV-A71 was also constructed (Fig. 1b).

CQ2014-86 shares the highest nucleotide identity with the Vietnamese strains. The nucleotide identities among them ranged from 97.5 to 99.9 %. The high similarity indicated that the strains had the same origin. Therefore, it is assumed that the virus was imported from neighboring countries and regions, possibly from Vietnam. Furthermore, the virus isolated from Xiamen City clustered with the strains from Taiwan, and the nucleotide identities between them ranged from 95.9 to 99.6 %, hence, the Xiamen strain may originate from Taiwan.

**Fig. 2:**
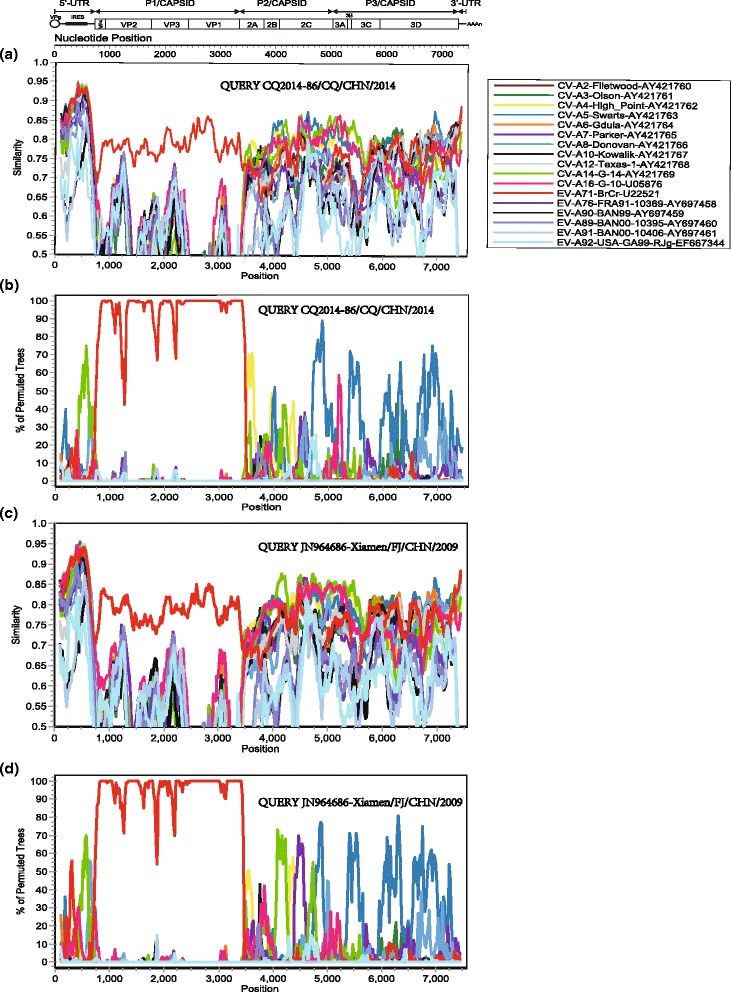
Similarity plot and bootscanning analyses of the whole genome of the EV-A strains. **a** Similarity plot and **b** bootscanning analysis. A sliding window of 200 nucleotides was used, moving in 20 nucleotide steps. The Chongqing strain CQ2014-86/CQ/CHN/2014 was used as the query sequence. **c** Similarity plot and **d** bootscanning analysis. A sliding window of 200 nucleotides was used, moving in 20 nucleotide steps. The Chongqing strain JN964686-Xiamen/FJ/CHN/2009 was used as the query sequence

The similarity plot and bootscanning analysis revealed that recombination exists between CQ2014-86 and other EV-A strains, such as CV-A4, CV-A5, CV-A14, and CV-A16 in the 5′UTR, and in the P2 and P3 regions (Fig. 2a and b). Also, the Xiamen strain has a similar recombinant pattern, thus reconfirming that recombination is a common phenomenon in enterovirus. (Fig. 2c and d).

## Discussion

The earliest subgenotype B5 of EV-A71 was isolated in Sarawak, Malaysia in 2000, and then in Yamagata, Japan in 2003 [7, 8]. The subgenotype B5, which is spread widely throughout the world, especially in Southeast Asia, has caused several disease outbreaks in Japan [7], Vietnam [9], Thailand [10], Singapore [11], Malaysia [8], and Taiwan (2008, 2012) [12, 13].

In China, most of EV-A71 belonged to subgenotype C4 [1, 2]. Frequent international travel, however, may result in some other subgenotypes being imported. This is the second report on importation of the subgenotype B5 of EV-A71 following the first, which occurred 5 years previously in Xiamen City in mainland China. Therefore, monitoring of imported subgenotypes EV-A71 should be strengthened.
